# Antidepressants, relapse-prevention medications and both combined to reduce alcohol-related hospitalizations in individuals with severe alcohol use disorder

**DOI:** 10.1007/s00406-025-01988-z

**Published:** 2025-03-12

**Authors:** Patrick Bach, Johan Franck, Jonas Hällgren, Härje Widing, Mika Gissler, Jeanette Westman

**Affiliations:** 1 Department of Clinical Neuroscience, Karolinska Institutet, Tomtebodavägen 18A, Stockholm, 17177 Sweden; 2 Department of Addictive Behavior and Addiction Medicine, Central Institute of Mental Health, Medical Faculty Mannheim, University of Heidelberg, Heidelberg, Germany; 3 Department of Data and Analytics, Finnish Institute for Health and Welfare, Helsinki, Finland; 4 Academic Primary Care Centre, Region Stockholm, Sweden; 5 Department of Molecular Medicine and Surgery, Karolinska Institutet, Stockholm, Sweden; 6 Department of Neurobiology, Care Sciences and Society, Karolinska Institutet, Stockholm, Sweden; 7 Department of Health Care Sciences, Marie Cederschiöld University, Stockholm, Sweden

**Keywords:** Alcohol use disorder, Antidepressants, Relapse-preventive medication, Hospitalization, Epidemiology, Register-based study

## Abstract

Data on the comparative usefulness of medications commonly prescribed to individuals with alcohol use disorder (AUD) are scarce. This study compared the association between antidepressants, relapse-preventive AUD medication, both, and neither on the risk of subsequent alcohol-related hospitalization in individuals with severe AUD. This retrospective analysis of Swedish nationwide register data used Cox (primary analysis) and logistic (sensitivity analysis) regression models to assess the associations between medication exposure (antidepressants, AUD medication, both, neither) and risk of subsequent alcohol-related hospitalization. The analysis included data on 14,026 individuals who were admitted to the hospital for severe AUD between 2009 and 2020. Antidepressants were not significantly associated with a lower risk of subsequent alcohol-related hospitalization (hazard ratio [HR] = 0.94, 95% confidence interval [CI] = 0.82–1.08), but AUD medication was (HR = 0.61, 95% CI = 0.54–0.69), as were antidepressants plus AUD medication (HR = 0.63, 95% CI = 0.45–0.87) (reference: exposure to neither). Pairwise comparisons showed that AUD medication was associated with a lower risk of hospitalization than antidepressants (HR = 0.65, 95% CI = 0.54–0.78). Antidepressants alone were associated with a higher risk than antidepressants plus AUD medication (HR = 1.50, 95% CI = 1.05–2.15). The sensitivity analysis confirmed the association between AUD medication and lower hospitalization risk. Antidepressant monotherapy was not associated with a lower risk of subsequent alcohol-related hospitalization, but relapse-preventive medication for AUD was, both alone and in combination with antidepressants. These findings support the use of relapse-preventive medication to reduce hospitalization risk in individuals with severe AUD and raise questions about the benefit of antidepressant monotherapy for this purpose.

**Supplementary Information:**

The online version contains supplementary material available at 10.1007/s00406-025-01988-z.

## Introduction

Alcohol use disorder (AUD) is highly prevalent and associated with significant impairment in the mental and physical health of affected individuals [9]. Pharmacotherapeutic options for the treatment of AUD include naltrexone, disulfiram, nalmefene, and acamprosate [15, 28]. Beyond the approved medications, many patients with AUD receive antidepressants to treat common symptoms such as insomnia, dysphoria, and anxiety, but also with the aim of supporting abstinence [5]. Substantially more patients with AUD (with and without comorbid depression) receive antidepressants than relapse-preventive medication. Whereas an estimated 10–60% of patients with AUD receive antidepressants [8, 18], 3–25% receive relapse-preventive AUD medication [6, 20, 23, 27]. Still, the evidence for antidepressants in treating AUD is far from conclusive. Two early meta-analyses reported that antidepressants (classes combined) significantly decreased depression severity but had no significant effect on alcohol use in patients with AUD [13, 21]. Similarly, a subsequent meta-analysis reported that selective serotonin reuptake inhibitors (SSRIs) reduced depression severity but had no effect on alcohol use in patients with AUD [26]. A Cochrane review concluded that the evidence that antidepressants (classes combined) reduce depression severity is of low quality, and the evidence that they improve alcohol abstinence in individuals with comorbid AUD and depression is of moderate quality [1]. Although antidepressants are frequently prescribed to patients with AUD, their efficacy in individuals with severe AUD, alone or in combination with approved AUD medication, remains unclear. To address this question, we compared the association between exposure to antidepressants, exposure to relapse-preventive medication for AUD, overlapping exposure to both, and exposure to neither on the risk of subsequent alcohol-related hospitalizations in individuals with severe AUD.

## Methods

### Data sources

The data for this nationwide cohort study were collected from various registers maintained by Statistics Sweden and the National Board of Health and Welfare (Table 1). The Total Population Register (https://www.scb.se/vara-tjanster/bestall-data-och-statistik/register/registret-over-totalbefolkningen-rtb) was used to identify Swedish residents, as well as their age and sex. The Longitudinal Integrated Database for Health Insurance and Labour Market Studies (LISA) (https://www.scb.se/en/services/ordering-data-and-statistics/register/longitudinal-integrated-database-for-health-insurance-and-labour-market-studies-lisa/) provided information on education level and cohabitation status. The National Patient Register (https://www.socialstyrelsen.se/statistik-och-data/register/patientregistret/) was used to obtain diagnostic information from inpatient and outpatient care. Prescription information came from the National Prescription Register (https://www.socialstyrelsen.se/statistik-och-data/register/lakemedelsregistret/). The National Cause of Death Register (https://www.socialstyrelsen.se/statistik-och-data/register/dodsorsaksregistret/) was used to determine the date of death for deceased individuals. Register data of individuals from the different sources were linked using the personal identification number (PIN) that is unique to each Swedish resident. The study was approved by the Swedish Ethical Review Authority (decision id 2019 − 00516). No informed consent was required since no registered person was contacted, and only anonymized data was used in this register-based study.

**Table 1:** Description of the time-invariant and time-variant covariates for which the statistical models were adjusted

Covariate	Levels	Description	Source
***Time invariant***			
Age	18–25; 26–35; 36–45; 46–55, 56–64	Age at index	Total Population Register
Sex	male; female	Sex at index	Total Population Register
Education	Primary or less, Secondary, University	Education level at index	The longitudinal integrated database for health insurance and labour market studies (LISA)
Hospitalizations	0; 1–2; 3–5; ≥6	Registered number of previous alcohol-related (F10) hospitalizations prior to study entry (since 1997)	The National Patient Register
Cohabitation	living single; living with another	Cohabitation status at index (living with another was defined as individuals being married, or living with children under the age of 18)	The longitudinal integrated database for health insurance and labour market studies (LISA)
***Time variant***			
Medication	Antidepressants; AUD medication; Both combined; Neither of both medication	Exposure to medication	The National Prescription Register

AUD = Alcohol Use Disorder

### Study population

According to international guidelines [11, 14, 22], people with an alcohol dependence according to the International Statistical Classification of Diseases and Related Health Problems, 10th Edition (ICD-10), corresponding to a severe AUD, are the main target group for pharmacological relapse prevention. The study population therefore consisted of individuals who received an inpatient diagnosis of an alcohol dependence, corresponding to a severe AUD, between 2009 and 2020. Severe AUD, i.e. alcohol dependence, was defined based on the ICD-10 code F10.2. The date of the individual’s first hospitalization with diagnosis code F10.2 (i.e. alcohol dependence) as the main diagnosis during the observational period was used as the individual’s index date, that is, the date on which a one-year follow-up period started. Additionally, individuals were only included if they were between 18 and 64 years old and resided in Sweden at the time of inclusion. To avoid potential bias, we excluded individuals with a history of prescriptions for relapse-preventive medications for AUD, antidepressants, or diagnoses of depressive disorders (ICD-10 codes F32, F33, F34.1-F34.9, F39) or anxiety disorders (ICD-10 codes F40-F41) one year prior to inclusion. The cohort included a total of 14,026 individuals. The follow-up period continued until 365 days after the index date or until the individual was hospitalized with a main diagnosis of a mental and behavioural disorder due to use of alcohol (ICD-10 code F10.0-F10.9), turned 65 years old, emigrated from Sweden, or died, whichever occurred first. We chose the length of the follow-up period because of high relapse rates after initial inpatient treatment. Previous research shows that most individuals return to substance use within the first three months following treatment, and fewer than 30% are abstinent one year after treatment [24].

### Exposure to medication

Exposure to medication was divided into four categories: exposure to antidepressants, exposure to relapse-preventive medication for AUD, overlapping exposure to both, and exposure to neither. Exposure to antidepressant and relapse-preventive medications were defined using data on prescription and dispensing of each medication from the National Prescribed Drug Register. These data included the prescription date, the dispensing date, the anatomical therapeutic chemical (ATC) code, and information on the amount of dispensed antidepressants and relapse-preventive medication for AUD. We included AUD medication (e.g. disulfiram, acamprosate, naltrexone, nalmefene) (ATC code N07BB) and antidepressants from the following groups: SSRIs (ATC N06AB), serotonin and norepinephrine reuptake inhibitors or SNRIs (e.g. venlafaxine, milnacipran, duloxetine)/ serotonin–norepinephrine–dopamine reuptake inhibitors or SNDRIs (e.g. nefazodone)/ tetracyclic antidepressants (e.g. mirtazapine)/ antidepressants with other modes of action (e.g. esketamine, tianeptine, bupropion, agomelatine, trazodone) (ATC N06AX), tricyclic antidepressants or TCAs (ATC N06AA), and monoamine oxidase inhibitors or MAOIs (ATC N06AF and N06AG). Exposure periods were calculated separately for antidepressants and AUD medication. The start of an exposure was set to the date the medication was dispensed to the individual, i.e., the date the prescription was filled. To define the end of an exposure, we first identified the total amount of the specific drug that was dispensed. We then used the defined daily dose (DDD) for AUD medication and the recommended therapeutic starting dose for antidepressants (see Table S1 in the supplement) to define expected daily medication intake and determine the duration and end date of an exposure. For AUD medication, the start and end of the exposure were determined as follows:

$$\:Start\:date\:=Dispense\:date$$

$$\eqalign{& \>End\>date\> = \> \cr & Dispense\>date + {{Amount\>of\>dispensed\>medication\>\left( {mg} \right)\>} \over {Defined\>Daily\>Dose\>(mg/day)}}\> \cr} $$

For antidepressants, the duration of the exposure was calculated by dividing the total amount of medication dispensed in mg by the recommended starting dosage for each medication (see Table S1 in the supplement). We also incorporated a 15-day delay in the start date of the exposure to antidepressants, because previous studies show that the therapeutic effects of antidepressants often have a delayed onset and that the maximum improvement occurs during the first 2 weeks after treatment initiation [19, 25]. For antidepressants, the start date of the exposure period was hence determined as follows:

$$\:Start\:date\:=Dispense\:date+15\:days$$

$$\eqalign{& \>End\>date\> = \cr & Dispense\>date + {{Amount\>of\>dispensed\>medication\>\left( {mg} \right)} \over {Recomended\>starting\>dosage\>\left( {{{mg} \over {day}}} \right)}} \cr} $$

When two or more exposure periods to medication with the same ATC code overlapped, we assumed stockpiling, that is, that the individual obtained additional medication before the previous supply was exhausted. The exposure period was extended by the number of days that the overlapping period lasted. When remaining doses from previous prescriptions could bridge the period to the next time the medication with the same ATC code was dispensed, we considered exposure to be continuous. Exposures to the same ATC code with gaps of up to five days in between were also considered to be continuous. Overlapping exposure to antidepressants and medication for AUD was defined as simultaneous exposure to both medication groups.

### Measures

The outcome measure was the first inpatient hospitalization during the follow-up period with the main diagnosis of an alcohol-related disorder, including ICD-10 codes F10.0 alcohol intoxication, F10.1 alcohol abuse, F10.2 alcohol dependence, F10.3 alcohol withdrawal, F10.4 alcohol withdrawal syndrome with delirium, F10.5 alcohol-induced psychotic disorder, F10.6 alcohol-related amnesic syndrome, F10.7 alcohol-associated residual and delayed-onset psychotic disorder, F10.8 other mental and behavioral disorders due to alcohol, and F10.9 unspecified mental and behavioral disorder due to alcohol.

### Data analysis

We used a Cox regression model as our primary statistical model to calculate hazard ratios (HRs) and 95% confidence intervals (CIs). The outcome was the first alcohol-related hospitalization during the follow-up period. In the model, the time-varying covariate was medication exposure (exposure to antidepressants, exposure to AUD medication, overlapping exposure to both, or exposure to neither) and follow-up time was used as the underlying time scale. To control for potential bias by differences in clinical and sociodemographic baseline characteristics, the Cox regression model was adjusted for sex, age, number of previous alcohol-related hospitalizations, cohabitation status, and education level at baseline (time-invariant covariates) (see Table 1 for a detailed description of covariates). To ensure the suitability of the Cox regression model, the proportional hazards (PH) assumption was checked by visual inspection of the scaled Schoenfeld residuals versus time with a smoothed curve superimposed to assess any trends.

### Sensitivity analysis

To validate the robustness of the primary Cox regression model, we constructed a logistic regression model to assess the association between medication exposure and hospitalization due to AUD, independent of the specific time point of hospitalization. The logistic regression model tested the association between medication exposure and risk of hospitalization during the one-year follow-up period as a dichotomous outcome. In the logistic regression model, exposure on the index date was used to define exposure groups. Here we considered a three-day grace period. That is, individuals not dispensed medication on the index date or the following three days were counted as unexposed. A three-day grace period was chosen to accommodate a delayed dispensing, e.g. due to closure of pharmacies on weekends and public holidays. The three day period was also selected based on the data indicating that most of the dispenses occurred within this timeframe, while only a small increase of about 15% was observed for the following 4 days (i.e. first week). A three day period was also chosen to reduce the likelihood of inter-current events, which could bias the observed associations between medication exposure and hospitalization risk. Individuals who were hospitalized again within three days after index date (*n* = 349) or died (*n* = 484) during the follow-up period of one year were excluded from the analysis, yielding a sample size of 13,193 individuals. Based on their exposure on the index date, 303 individuals were assigned to the group exposed to antidepressants, 1,516 to the group exposed to AUD medication, 188 to the group exposed to both, and 11,186 individuals to the group exposed to neither antidepressants nor AUD medication. The logistic regression model was adjusted for the same covariates as the Cox regression model. To confirm the robustness of the results against different durations of the grace period, models considering a 0 and 7 day grace period were set-up and tested.

## Results

The study included data on 14,026 individuals with a diagnosis of severe AUD. The majority were male (*n* = 11,146; 79.5%), and the mean age of individuals in the study cohort was 49.7 years (SD = 10.7 years) (Table 2). During follow-up, 1,780 individuals (12.7%) were dispensed antidepressants at least once, 3,287 (23.4%) were dispensed AUD medication at least once, and 571 (4.1%) were dispensed antidepressants and AUD medication during overlapping periods. Most patients who were dispensed antidepressants received either SSRIs, SNRIs, or SNDRIs (Table 2). The median duration of exposure was 108 days for antidepressants (mean = 143.6 days), 100 days for AUD medication (mean = 107.4 days), and 78 days for overlapping exposure to both (mean = 90.3 days). During the one-year follow-up, a total of 5,260 individuals (37.5%) had an alcohol-related hospitalization and 484 (3.5%) died. The average time until first re-hospitalization was 124 days (median = 96 days) and longer in individuals exposed to either AUD medication (mean = 158 days, median = 146 days) or antidepressants (mean = 126 days, median = 104 days) or both combined (mean = 132 days, median = 104 days), compared to individuals that were exposed to neither of both (mean = 121 days, median = 93 days).

**Table 2:** Description of the study population (*N* = 14,026), including all residents aged 18–64 years, living in Sweden with first registered hospitalization, due to severe AUD (ICD-10: F10.2) between 2009–2020

Variable	*N* (%)
**Sex**	
Female	2880 (20.5)
**Age at study entry**	
Mean (Standard Deviation)	49.70 (10.7)
**Education level at study entry**	
University	1706 (12.2)
Secondary education	7501 (53.5)
Primary education	4624 (33.0)
Unknown/No primary education	195 (1.4)
**Cohabiting at study entry**	
Yes	2847 (20.3)
**Comorbid SUD (F11-F16**,** F18-F19) in the year prior to study entry**	
Yes	1713 (12.2)
**Number of alcohol-related hospitalizations prior to study entry**	
≥ 6	1967 (14)
3–5	1646 (11.7)
1–2	3597 (25.6)
0	6816 (48.6)
**Antidepressant exposure during follow-up**	
Yes	1780 (12.7)
Mean duration of exposure in days (Standard Deviation)	143.6 (111.6)
Median duration of exposure in days	108
**Exposure to different antidepressant classes**	
SSRI (ATC: N06AB)	1019 (57.2)
TCA (ATC: N06AA)	111 (6.2)
MAOI (ATC: N06AF & N06AG)	1 (0.1)
Others– SNRI, SSNRI, SNDRI (ATC: N06AX)	1001 (56.2)
**Exposure to antidepressant and AUD medication concurrently during follow-up**	
Yes	571 (4.1)
Mean duration of exposure in days (Standard Deviation)	90.3 (78.4)
Median duration of exposure in days	78
**AUD medication exposure during follow-up**	
Yes	3287 (23.4)
Mean duration of exposure (Standard Deviation)	107.4 (82.7)
Median duration of exposure	100
**Exposure to different AUD medication classes**	
Acamprosate (ATC: N07BB03)	1226 (37.3)
Naltrexone (ATC: N07BB04)	871 (26.5)
Disulfiram (ATC: N07BB01)	1925 (58.6)
Nalmefene (ATC: N07BB05)	13 (0.4)
**Alcohol-related hospitalization(s) during follow-up**	
Yes	5260 (37.5)
**Death during follow-up**	
Yes	484 (3.5)

Legend: AUD = Alcohol Use Disorder, SUD = Substance Use Disorder, SSRI = Selective Serotonin Reuptake Inhibitors, SNRI = Selective Noradrenaline Reuptake Inhibitors, SSNRI = Selective Serotonin-Noradrenaline Reuptake Inhibitors, SNDRI = Selective Noradrenaline-Dopamine Reuptake Inhibitors, TCA = Tricyclic Antidepressants, MAOI = Monoamine Oxidase Inhibitors

**Fig. 1:**
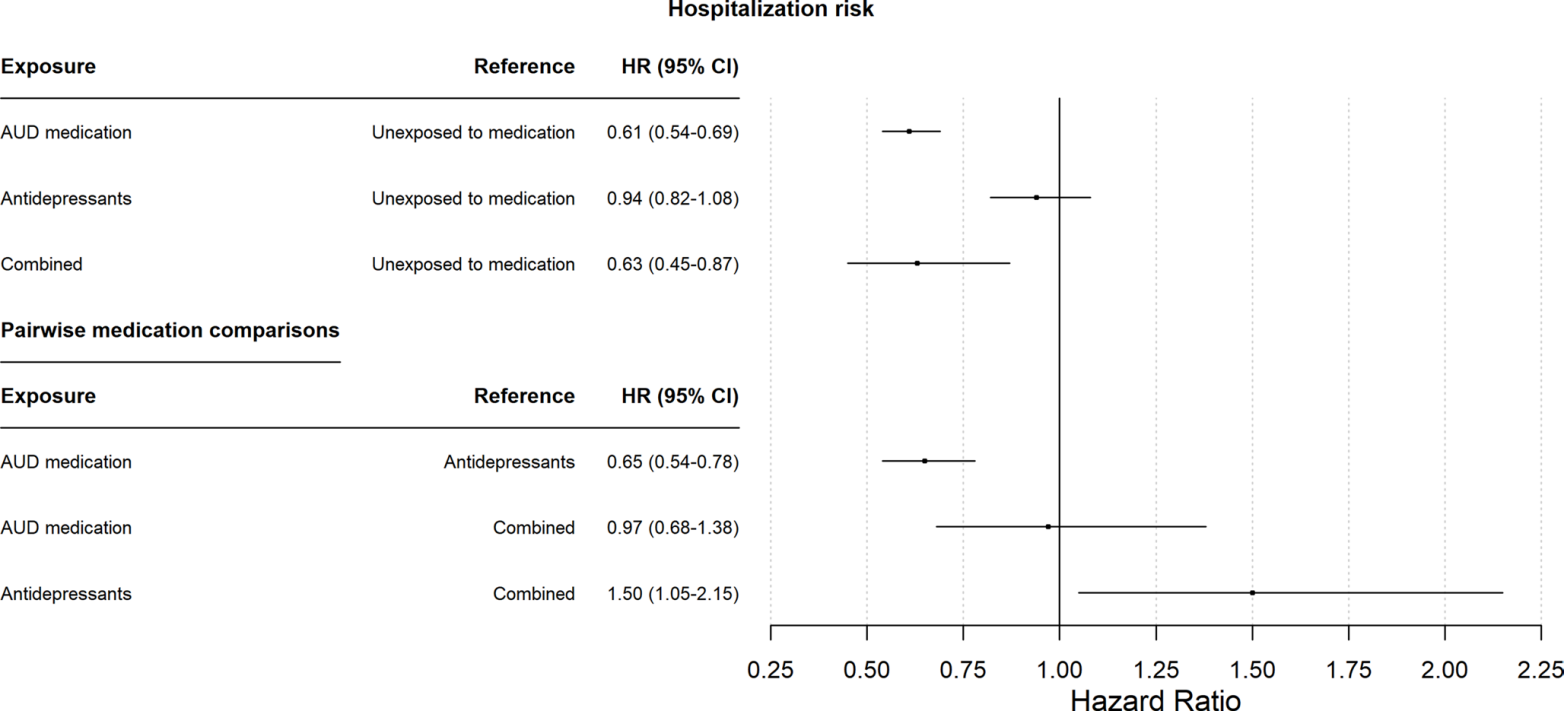
Adjusted hazard ratios (HRs) and 95% confidence intervals (CIs), derived from the Cox regression model, for the association between time to first alcohol-related hospitalization and exposures to either antidepressants, relapse-preventive alcohol use disorder (AUD) medication or both concurrently compared to exposure to neither antidepressants nor AUD medication, and pairwise comparison between medication categories. The model was adjusted for age, sex, education level, number of previous alcohol-related hospitalizations and cohabitation status (see Table 1 for details)

The results of the primary analysis using a Cox regression model showed that exposure to AUD medication was associated with a lower risk of subsequent alcohol-related hospitalization than exposure to neither AUD medication nor antidepressants (hazard ratio [HR] = 0.61, 95% confidence interval [CI] = 0.54–0.69) (Fig. 1). Similarly, overlapping exposure to both medications was associated with a lower risk of subsequent alcohol-related hospitalization than exposure to neither (HR = 0.63, 95% CI = 0.45–0.87). However, exposure to antidepressants was not significantly associated with a lower risk of subsequent alcohol-related hospitalizations (HR = 0.94, 95% CI = 0.82–1.08).

Pairwise comparison of hospitalization risk by medication categories (Fig. 1) showed that risk of subsequent alcohol-related hospitalizations was significantly lower for exposure to AUD medication than exposure to antidepressants (HR = 0.65, 95% CI = 0.54–0.78). Furthermore, risk of alcohol-related hospitalization was significantly higher for exposure to antidepressants than for exposure to both medications (HR = 1.50, 95%CI = 1.05–2.15). No significant difference was observed between exposure to AUD medication and exposure to both medications (HR = 0.97, 95%CI = 0.68–1.38).

### Sensitivity analysis

**Fig. 2:**
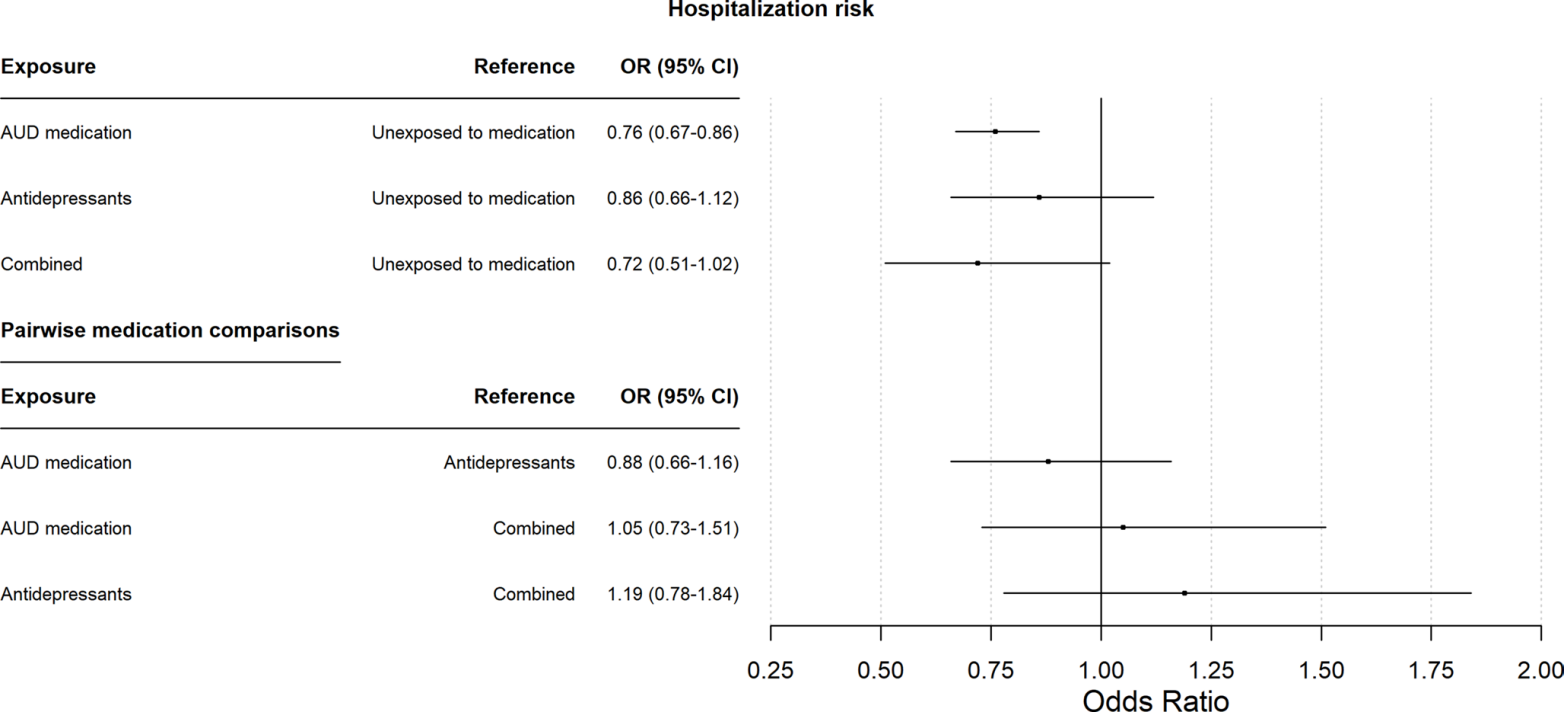
Odds ratios (ORs) and 95% confidence intervals (CIs), derived from the logistic regression model (sensitivity analysis), for the association between risk of any alcohol-related hospitalization during the one-year follow-up and exposures to either antidepressants, relapse-preventive alcohol use disorder (AUD) medication or both concurrently, compared to exposure to neither antidepressants nor AUD medication, and pairwise comparison between medication categories. The model was adjusted for age, sex, education level, number of previous alcohol-related hospitalizations and cohabitation status (see Table 1 for details)

In line with the findings of the primary analysis, the logistic regression model showed that exposure to AUD medication was associated with significantly lower odds of alcohol-related hospitalization during the one-year follow-up than exposure to neither antidepressants nor AUD medication (odds ratio ([OR] = 0.76, 95% CI = 0.67–0.86) (see Fig. 2). In contrast, exposure to antidepressants alone was not significantly associated with lower odds of alcohol-related hospitalizations (OR = 0.86, 95% CI = 0.66–1.12), and the same was true of overlapping exposure to both antidepressants and AUD medication (OR = 0.72, 95% CI = 0.51–1.02). Additional models that considered a grace period of 0 and 7 days to define exposure groups yielded similar results (see supplementary Table S2).

## Discussion

Exposure to relapse-preventive medication for AUD, alone or in combination with antidepressants, but not exposure to antidepressants alone, was associated with a lower risk of subsequent alcohol-related hospitalization than exposure to neither antidepressants nor AUD medications. Furthermore, the risk of subsequent alcohol-related hospitalization was lower for exposure to AUD medication and overlapping exposure to both medications than for exposure to antidepressants alone. The sensitivity analysis corroborated the robustness of the association between AUD medication and a lower risk of alcohol-related hospitalizations. These results suggest that dispensing AUD medication to individuals with severe AUD could reduce the risk of subsequent alcohol-related hospitalizations, whereas dispensing antidepressants alone could not.

A significant association between exposure to AUD medication and lower risk of alcohol-related hospitalization was also observed in a 2021 register-based study that used Swedish national data on all individuals with an alcohol-related diagnosis (i.e., all ICD-10 F10.0-F10.9 diagnoses) [12]. A further study also showed that dispensing medication for AUD to individuals with an alcohol-related hospitalization shortly after discharge from inpatient treatment was associated with a significantly lower risk of subsequent all-cause and alcohol-related hospitalizations in the following 30 days [3]. The results presented here are also in line with those of a meta-analysis that found that antidepressants did not reduce relapse risk in individuals with AUD who did not have mood disorders [26]. Although the findings of the current study do not support the hypothesis that antidepressant use reduces the risk of alcohol-related hospitalization in individuals with severe AUD, some studies indicate that antidepressant treatment may have beneficial effects on alcohol use in individuals with AUD and comorbid depression [1, 10]. However, other studies have not found that antidepressants have significant effects on either alcohol use [13, 21] or remission and abstinence [16] in those with comorbid depression and AUD. For example, a retrospective study of medical records found that discharging patients with AUD and comorbid depression with a prescription for an antidepressant was not associated with reduced alcohol-related readmissions [4]. The lines of evidence suggest that antidepressants might not reduce alcohol use and relapse risk early in treatment, even though these effects seem to be important when targeting AUD.

We also found that in individuals with severe AUD without comorbid depression or anxiety, overlapping exposure to antidepressants and AUD medication was associated with a lower risk of alcohol-related hospitalizations than exposure to antidepressants only. This finding supports the notion that if antidepressants are prescribed to reduce AUD symptoms and hospitalization risk in individuals with severe AUD who do not have depression or anxiety, then AUD medication should also be prescribed. However, the more rapid onset of effects from AUD medication than antidepressants [25] is one possible explanation for the presented findings. To mitigate potential bias due to the slower onset of antidepressant effects, we used a 15-day delay between the date antidepressants were dispensed and the start of the exposure period in our primary analysis. In addition, we constructed a logistic regression model to test associations between medication exposure and risk of alcohol-related hospitalizations independent of exposure durations. Consistent with the results of the primary analysis, the logistic regression confirmed the significance of the association between exposure to AUD medication and the risk of an alcohol-related hospitalization, while no significant association between exposure to antidepressants and the risk of an alcohol-related hospitalization was observed. Results on the comparisons between the different medication groups were descriptively similar to the primary analysis, but failed to yield significance, potentially because of the lower power and sensitivity than in the primary model. In contrast to the primary analysis, the association between exposure to antidepressants and AUD medication combined showed no significant association with hospitalization risk. Limited size of the medication groups of the logistic regression model could help explain why the associations did not surpass the threshold for significance, which is also illustrated by the large confidence intervals. Still, the consistent results of the primary and secondary analysis on exposure to AUD medication and antidepressants support the robustness of the main findings.

A portion of the study population received antidepressant prescriptions even though we excluded individuals diagnosed with depression and anxiety disorders with most individuals receiving either an SSRI or an antidepressant from the group of SNRIs, SSNRIs and SNDRIs. Thus the results can be considered representative for mainly these classes of antidepressants. In contrast, although all individuals in the study population likely had an indication for receiving AUD medication, only about a quarter received prescriptions for such medication, suggesting that AUD medications are underutilized in this population despite their efficacy [17]. This pattern of prescription rates has also been observed in previous studies [8, 18]. The present results suggest that AUD medication is still an underused pharmacological treatment option for individuals with severe AUD and that expanding their use may significantly improve outcomes in affected individuals.

### Strengths and limitations

A strength of the study is that it provides information relevant to a main target group for relapse-prevention interventions, individuals with an alcohol dependence, corresponding to a severe AUD, who require hospital treatment for their AUD. The selection of the study population and the index date, based on a main diagnosis of severe AUD and first hospitalization for this diagnosis during the study period, means that the results are primarily generalizable to treatment-seeking individuals with severe AUD. The findings might be less relevant to populations that do not seek treatment or people with mild to moderate AUD that do not meet the diagnosis of an alcohol dependence according to the ICD-10. Still, a previous register-based study that included all individuals with AUD in Sweden (ICD-10 codes F10.0-F10.9) also showed a significant association between exposure to AUD medications and reduced hospitalization risk, which indicates that the presented results might translate to a broader population of individuals with varying AUD severity [12].

Register data is– by its nature– retrospective and observational, so the effects observed in the study are associative, and conclusions should be drawn with caution. Information on daily alcohol use and abstinence are not included in the registries, and alcohol-related hospitalizations were therefore used as the outcome. Nevertheless, alcohol-related hospitalizations can be considered an important health-related outcome.

A limitation of the study is the lack of data to externally validate whether the calculated exposures match true exposures. In theory, similar prescriptions with different dosing schedules could have contributed to different true exposures, especially for antidepressants. To mitigate the potential for over- and underestimating exposure duration, the recommended mean therapeutic dose was used as the reference dose. Additional analyses, which used the maximum recommended dose for each type of antidepressant as the basis for calculating the duration of exposure (i.e. to test an extreme scenario assuming short exposures), confirmed the results of the primary analysis. The results of the primary analyses were also confirmed in the sensitivity analysis that used a logistic regression model.

The one-year follow-up period might have limited the capacity to detect long-term effects but was also a strength of the study. Evidence suggests that the proportion of patients who return for alcohol-related inpatient care is largest in the first year of follow-up, which suggests that this period is most informative [2]. By limiting the sample to individuals with the diagnosis of a severe AUD and exclusion of individuals with a prior diagnosis of a depression or anxiety disorder, as well as the focus on individuals that received a medication dispense shortly after in-patient treatment due to AUD, we aimed to focus on a population that was likely to receive the respective medication under investigation, due to their AUD. It should be noted that our analyses didn’t specifically exclude individuals with other mental health diagnoses, such as borderline personality disorder or posttraumatic stress disorder, which might also be associated with receiving (off-label) prescriptions for either antidepressants or AUD medication. Still, the number of such individuals in the sample population was rather low (*n* = 97) and the significance of the results of the primary analyses remained unchanged after exclusion of these individuals (for details see supplementary Table S3).

Because of the low number of individuals who received different classes of antidepressants, class-specific analyses were not feasible. Still, most meta-analyses suggest that effects of different antidepressant classes on alcohol use and relapse risk do not show robust differences [1, 7, 10, 13]. In addition, the subgroups exposed to antidepressants, AUD medication, both or neither were quite asymmetrical in size. Thus, limited power could help explain why the association between antidepressants and hospitalization risk did not surpass the threshold for significance.

### Conclusions

The results of this study show that exposure to AUD medication is associated with a lower risk of subsequent alcohol-related hospitalizations in individuals with severe AUD than exposure to antidepressants or exposure to neither of the two medication classes. This supports the recommendation to offer relapse-preventive AUD medication to individuals with severe AUD and raises questions about the benefit of dispensing antidepressants to prevent alcohol-related hospitalizations in individuals with severe AUD.

## Electronic supplementary material

Below is the link to the electronic supplementary material.

Supplementary Material 1
