# Supplementary material for: Antidepressants, relapse-prevention medications and both combined to reduce alcohol-related hospitalizations in individuals with severe alcohol use disorder
Source: Eur Arch Psychiatry Clin Neurosci. 2025 Mar 12;276(2):761–70. doi: 10.1007/s00406-025-01988-z (PMC12953294; doi:10.1007/s00406-025-01988-z)
Supplement: Supplementary file 1 — Supplementary Material 1 [file 406_2025_1988_MOESM1_ESM.docx]

# Supplements

**Dispensing of antidepressants and relapse-preventive medication in reducing alcohol-related hospitalizations in treatment-seeking individuals with severe alcohol use disorder**

Bach et al.

**Content**

**Supplementary Tables**

- **Supplementary Table S1.** Depiction of the recommended starting dose for different antidepressants
- **Supplementary Table S2.** Results of the additional sensitivity analyses considering a zero and seven day grace period to define exposure groups.
- **Supplementary Table S3.** Results of the primary analysis for the sub-sample of individuals without prior diagnosis of a borderline personality disorder (BPD) or a posttraumatic stress disorder (PTSD)

**Table S1.** Depiction of the recommended starting dose for different antidepressants, according to Swedish medical product information, which were used to estimate expected daily medication intake and exposure duration (see Methods for detailed description).

| **Medication** | **Daily dose considered for determining exposure duration (mg)** |
| --- | --- |
| Citalopram | 20.0 |
| Escitalopram | 10.0 |
| Fluoxetine | 20.0 |
| Fluvoxamine | 50.0 |
| Paroxetine | 20.0 |
| Sertraline | 50.0 |
| Desvenlafaxien | 25.0 |
| Duloxetine | 30.0 |
| Levomilnacipran | 20.0 |
| Milnaciprane | 12.5 |
| Venlafaxine | 37.5 |
| Agomelatine | 25.0 |
| Bupropion | 200.0 |
| Mirtazapine | 15.0 |
| Nefazodone | 200.0 |
| Trazodone | 100.0 |
| Vilazodone | 10.0 |
| Vortioxetine | 10.0 |
| Amitriptyline | 25.0 |
| Amoxapine | 25.0 |
| Clomipramine | 25.0 |
| Desipramine | 25.0 |
| Doxepin | 25.0 |
| Imipramine | 25.0 |
| Maprotiline | 25.0 |
| Nortriptyline | 25.0 |
| Protriptyline | 10.0 |
| Trimipramine | 25.0 |
| Isocarboxazid | 10.0 |
| Fenelzine | 15.0 |
| Tranylcypromine | 10.0 |
| Dosulepin | 75.0 |
| Esketamine | 56.0 |
| Lofepramine | 75.0 |
| Mianserine | 30.0 |
| Moclobemid | 300.0 |
| Opipramol | 200.0 |
| Oxitriptan | 100.0 |
| Reboxetine | 8.0 |
| Tianeptine | 37.5 |

Legend: mg = milligramms

**Table S2.** Results of the additional sensitivity analyses considering a zero and seven day grace period to define exposure groups. That is, individuals not dispensed medication on the index date or the following 0/7 days were counted as unexposed. Odds ratios (ORs) and 95% confidence intervals (CIs), derived from the logistic regression model (sensitivity analysis), for the association between risk of any alcohol-related hospitalization during the one-year follow-up and exposures to either antidepressants, relapse-preventive alcohol use disorder (AUD) medication or both concurrently, compared to exposure to neither antidepressants nor AUD medication, and pairwise comparison between medication categories. The model was adjusted for age, sex, education level, number of previous alcohol-related hospitalizations and cohabitation status.

| **Grace period of 0 days** |  |  |
| --- | --- | --- |
| **Exposure** | **Reference** | **OR (95% CI)** |
| AUD medication | Unexposed to medication | 0.64 (0.54-0.77) |
| Antidepressants | Unexposed to medication | 0.78 (0.55-1.12) |
| Combined | Unexposed to medication | 0.76 (0.49-1.18) |
|  |  |  |
| **Grace period of 7 days** |  |  |
| **Exposure** | **Reference** | **OR (95% CI)** |
| AUD medication | Unexposed to medication | 0.78 (0.70-0.88) |
| Antidepressants | Unexposed to medication | 0.84 (0.67-1.07) |
| Combined | Unexposed to medication | 0.77 (0.56-1.06) |

**Table S3.** Results of the primary analysis for the sub-sample of individuals without prior diagnosis of a borderline personality disorder (BPD) or a posttraumatic stress disorder (PTSD) (n=97 individuals with registered diagnosis of either PTSD or BPD were removed from the analysis population, leaving N=13,929 individuals). Adjusted hazard ratios (HRs) and 95% confidence intervals (CIs), derived from the Cox regression model for the association between time to first alcohol-related hospitalization and exposures to either antidepressants, relapse-preventive alcohol use disorder (AUD) medication or both concurrently compared to exposure to neither antidepressants nor AUD medication, and pairwise comparison between medication categories. The model was adjusted for age, sex, education level, number of previous alcohol-related hospitalizations and cohabitation status.

| **Exposure** | **Reference** | **HR (95% CI)** |
| --- | --- | --- |
| AUD medication | Unexposed to medication | 0.61 (0.54-0.69) |
| Antidepressants | Unexposed to medication | 0.94 (0.82-1.08) |
| Combined | Unexposed to medication | 0.61 (0.44-0.86) |
|  |  |  |
| **Pairwise medication comparisons** | |  |
| **Exposure** | **Reference** | **HR (95% CI)** |
| AUD medication | Antidepressants | 0.65 (0.54-0.78) |
| AUD medication | Combined | 0.99 (0.70-1.42) |
| Antidepressants | Combined | 1.53 (1.06-2.20) |
